# Editorial: Immunometabolic response of natural products in inflammation and cancer

**DOI:** 10.3389/fimmu.2023.1335510

**Published:** 2023-11-17

**Authors:** Wen Tan, Dong-Hua Yang, Zhangfeng Zhong

**Affiliations:** ^1^ School of Pharmacy, Lanzhou University, Lanzhou, China; ^2^ New York College of Traditional Chinese Medicine, Mineola, NY, United States; ^3^ Macao Centre for Research and Development in Chinese Medicine, State Key Laboratory of Quality Research in Chinese Medicine, Institute of Chinese Medical Sciences, University of Macau, Macao, Macao SAR, China

**Keywords:** immunometabolism, natural products, cancer, inflammation, traditional chinese medicine

Immunometabolism is an interwoven conception in modern pharmacology research. New technologies, new discoveries, and new ideas are being added to make this conception more mature and richer. The concept of immunometabolism is particularly significant in basic and clinical research on inflammation and cancer. Since most of the drugs are derived from natural products, research on immunometabolism for natural products is also valuable for new drug discovery.

This Research Topic collects several interesting articles. Norbergenin, a polyphenolic gallic acid derivative, frequently seen in the use of ethnobotanical medicine. Li et al. explored the mechanism of impairing LPS-induced signaling in macrophages and pointed out the role of glycolysis and TCA cycle intermediates. Because of its anti-inflammatory potential, this study is a typical study of immunometabolism, which is particularly inspiring for researchers who study the LPS stimulated macrophages in inflammation. Meanwhile, a combination of proteomic analysis, metabolomic analysis and bioinformatic analysis in one study is also worth learning from the comprehensive verification. As a natural substance, creatine is an indispensable organic compound utilized in various biological activities. The human body can obtain creatine from a variety of natural sources, such as fish and meat, and is taken up by the creatine transporter. Not only that, creatine itself also has an important metabolic role, creatine metabolism. Maintaining creatine homeostasis is crucial for proper biological and physiological conditions. Hence, the contribution of creatine to immunity is worth further study. Peng and Saito et al. demonstrated that creatine increased intracellular ATP levels, regulated immunological activities of macrophages, and strengthened CD8+ T cell-based anti-tumor immunity. In addition, creatine can enhance immunity by modulating macrophages, which also helps to sort out the relationship between immunological function and metabolic characteristics. In addition to the above, the immunomodulation and escape mechanisms in tumors, as well as the anti-tumor immunomodulatory activities of some representative active ingredients of traditional Chinese medicine were reviewed and discussed by Yang et al. Additionally, a heat shock protein heat-shock-protein family A (Hsp70) member 5 (HSPA5) was reviewed by Li et al. to explore its therapeutic and prognostic significance and prospect in cancers, indicating the significance in cancer treatment by targeting HSPA5 expression via natural products in the future.

In summary, these inspiring studies will help elucidating the role of immunometabolism of natural products, and promote novel drug discovery from natural products.

## Author contributions

WT: Conceptualization, Writing – original draft, Writing – review & editing. DY: Conceptualization, Writing – review & editing. ZZ: Conceptualization, Writing – review & editing, Writing – original draft.

